# Identifying Cardio-Metabolic Subtypes of Prediabetes Using Latent Class Analysis

**DOI:** 10.3390/medsci13040243

**Published:** 2025-10-25

**Authors:** Gulnaz Nuskabayeva, Yerbolat Saruarov, Karlygash Sadykova, Mira Zhunissova, Nursultan Nurdinov, Kumissay Babayeva, Mariya Li, Akbota Zhailkhan, Aida Kabibulatova, Antonio Sarria-Santamera

**Affiliations:** 1 Department of Special Clinical Disciplines, Medical Faculty, Khoja Akhmet Yassawi International Kazakh-Turkish University, Bekzat Sattarkhanov Street No. 29, Turkistan 161200, Kazakhstan; 2 Department of Fundamental Sciences, Medical Faculty, Khoja Akhmet Yassawi International Kazakh-Turkish University, Bekzat Sattarkhanov Street No. 29, Turkistan 161200, Kazakhstan; 3 Department of Biomedical Sciences, Nazarbayev University School of Medicine, Astana 010000, Kazakhstan

**Keywords:** prediabetes, glucose metabolism, cardiovascular risk, latent class analysis, Kazakhstan, insulin resistance, β-cell function

## Abstract

**Background/Objectives:** Prediabetes (PreDM) is a heterogeneous condition, impacting hundreds of millions worldwide, associated with a substantially high risk of Type 2 Diabetes Mellitus (T2DM) and cardiovascular complications. Early identification of subgroups within the PreDM population may support tailored prevention strategies. **Methods:** We conducted a cross-sectional study using data from annual health check-ups of 419 university staff (aged 27–69) in Kazakhstan. Latent Class Analysis (LCA) was applied to identify subgroups of individuals with PreDM based on cardiovascular risk factors. Differences in glucose metabolism markers (fasting glucose, OGTT, HOMA-IR, HOMA-β) were compared across identified classes. **Results:** PreDM prevalence was 43.4%. LCA revealed four distinct classes: Class 1: healthy, low-risk individuals; Class 2: overweight with moderate metabolic risk; Class 3: older, overweight individuals with high cardio-metabolic risk; and Class 4: obese, middle-aged to older individuals with very high cardio-metabolic risk. Significant differences were found in glucose metabolism profiles across the classes. IFG predominated in Class 1 (95%), while Classes 3 and 4 had higher rates of β-cell dysfunction and combined IFG/IGT patterns. HOMA-β differed significantly between classes (*p*  <  0.001), while HOMA-IR did not. **Conclusions:** PreDM is highly prevalent in this working-age Kazakh population and demonstrates marked heterogeneity. Based on easily obtainable cardiovascular risk factors, we have identified four subgroups with distinct glucose profiles that may inform personalized interventions. These distinct subgroups may require differentiated prevention strategies, moving beyond a one-size-fits-all approach.

## 1. Introduction

Prediabetes (PreDM) is a heterogeneous condition, impacting hundreds of millions worldwide, associated with a substantially high risk of Type 2 Diabetes Mellitus (T2DM) and cardiovascular complications, that represents an intermediate state of hyperglycemia defined by the elevation of plasma glucose levels above normal levels but below the criteria of T2DM diagnosis [1]. It is a high-risk condition impacting hundreds of millions worldwide, with considerable consequences for cardiovascular health and the advancement of diabetes [2]. The relevance of identifying PreDM is twofold: First, because it represents an increased risk of progression to T2DM, as up to 50% of individuals with PreDM will develop T2DM within 3–5 years [3], and globally it is expected that by 2050, 12.9% of the adult population will have PreDM [4]. Second, because PreDM is reversible through lifestyle modification programs based on healthier diets and increased levels of physical activity and/or medications [5]. As the prevalence of both PreDM and T2DM and the burden associated with these conditions continue to rise worldwide, there is a critical and urgent unmet need to identify those at the highest risk of developing T2DM and intervene to curb this epidemic [6].

The prevalence of PreDM is difficult to estimate, as a significant proportion of persons (ranging from 8% to 21%, depending on the criteria) are unaware of their condition. Previous data from Kazakhstan reported an 8% prevalence of DM and 1.9% of PreDM diagnoses [7]. The proportion of people tested in routine clinical conditions is low, and there is also a significant risk of incidence of T2DM among those not tested [8]. A recent update of the US Preventive Services Task Force reports that screening for PreDM and T2DM and offering or referring patients with PreDM to effective preventive interventions has a moderate net benefit [9].

As well as the lack of epidemiological data, there continues to be significant controversy [10] surrounding the precise characterization of PreDM [11]: how the relationships between impaired fasting glucose (IFG), impaired glucose tolerance (IGT), elevated glycosylated hemoglobin (HbA1c), insulin resistance (IR), and β-cell deficit interrelated. There is also controversy regarding its association with demographic, behavioral, clinical, and biochemical characteristics, primarily related to cardiovascular risk, as well as how ethnic and genetic differences may interact with the previous mentioned factors. In fact, PreDM is being recognized not as a singular entity but as a heterogeneous group characterized by diverse pathophysiology, risk of progression to T2DM, and aggregation of risk factors. Such heterogeneity in PreDM challenges the traditional view of it, as noted in a study by Tabák et al. (2012), pointing out the need for personalized medicine strategies [12]. Consequently, the “one-size-fits-all” prevention approach may not apply to different PreDM phenotypes, calling for a precision approach by matching subtypes with effective interventions that prevent T2DM [13].

The objective of this work is to describe the main characteristics of a sample of a diabetes-free general population in Kazakhstan, an ethnically diverse population; describe the prevalence of PreDM in this population and their main characteristics; and identify possible subtypes of PreDM using Latent Class Analysis (LCA) to identify unobserved associations between cardio-metabolic factors and glucose metabolism indexes. The rationale for analyzing simple cardiovascular risk indicators is because while oral glucose tolerance tests (OGTTs) and insulin-based measures such as HOMA-IR and HOMA-β provide valuable information about glucose metabolism, they are rarely obtained in routine clinical practice, especially in low-resource settings. Simpler cardiovascular risk indicators such as age, BMI, waist circumference, blood pressure, and lipid profiles are universally measured and inexpensive. The use of these widely available markers in this work may therefore offer a pragmatic approach to identifying clinically relevant subgroups of individuals with PreDM, having the advantage of being more easily implemented in real-world contexts.

## 2. Materials and Methods

The reporting of this study will follow the STROBE recommendations for observational studies [14]. This is a cross-sectional study with data obtained from annual medical check-ups from 2019 and 2020 of employees of Khoja Akhmet Yassawi International Kazakh-Turkish University (Turkistan, Kazakhstan). Data were collected at the Clinical Diagnostic Center of the university during routine health screenings of employees. Participants underwent standardized clinical assessments, including anthropometric measurements, blood sampling, and a 2 h oral glucose tolerance test (OGTT), between January 2019 and December 2020.

Eligible participants for this study were employees of the University aged 27–69 years who provided written informed consent. Exclusion criteria were having already been diagnosed with T1 or T2DM or kidney disease. After obtaining written informed consent form, demographic data, lifestyle, anthropometric, and biochemical laboratory data were obtained.

The primary outcome of this study was PreDM, defined by WHO criteria (FG: 6.1–6.9 mmol/L; OGTT 2 h glucose: 7.8–11.1 mmol/L). Secondary outcomes were glucose metabolism markers (FG, OGTT, HOMA-IR, HOMA-β). The exposures were cardiovascular risk factors used in LCA (continuous variables: age, BMI, waist circumference, blood pressure, total cholesterol, LDL, HDL, triglycerides).

Laboratory methods included the determination of fasting glucose levels; after a 2 h oral glucose tolerance test (OGTT), triglycerides (TG), total cholesterol (TC), high-density lipoprotein (HDL), and low-density lipoprotein (LDL) were measured. Blood sampling was carried out from the cubital vein after a 12 h fast. OGTT was performed with 75 g glucose solution. Plasma glucose levels were measured after 0 and 120 min. For PreDM, fasting glucose was taken as 6.1–6.9 mmol/L, after OGTT—7.8–11.1 mmol/L (WHO). Biochemical studies were determined in a biochemical analyzer (Cobas Integra-400 from Roche (Basel, Switzerland)). The laboratory determinations were carried out in the laboratory of the Clinical Diagnostic Center of Khoja Akhmet Yassawi International Kazakh-Turkish University.

HOMA-IR and HOMA-β were calculated and divided into 2 categories, namely IR and poor β-cell function. HOMA models were calculated as HOMA-IR = fasting insulin (lU/mL) × fasting glucose (mmol/L)]/22.5, and HOMA-β = [20 × fasting insulin (lU/mL)]/[fasting glucose (mmol/L) − 3.5]. IR was defined as values HOMA_IR ≥ 2.5 and poor β-cell function when HOMA-β ≤ 50 [15,16,17].

Kolmogorov–Smirnov and skewness tests were applied to assess normality of quantitative variables. The median and IQR were used since the continuous data was distributed non-normally. Categorical variables were described using frequency distribution. Pearson’s Chi-square test was used to compare characteristics between the healthy and the PreDM populations. To compare indices of groups divided based on HOMA indexes, we used Kruskal–Wallis tests.

Latent Class Analysis (LCA) for continuous variables was used to generate homogeneous groups of PreDM participants based on cardiovascular risk factors (age, BMI, waist and hip circumference, systolic and diastolic blood pressure, total cholesterol, LDL, HDL, and triglycerides). Bonferroni post hoc analysis was conducted to determine the inter-group significant differences. The appropriate number of classes were chosen based on the Akaike information criterion (AIC) and Bayesian information criterion (BIC) [18]. We then explored the cardio-metabolic characteristics of these LCA classes.

This study was performed in accordance with relevant guidelines/regulations and with the Declaration of Helsinki. This study was approved by Khoja Akhmet Yassawi International Kazakh-Turkish University (No. 27/2; 23 September 2019) and Nazarbayev University School of Medicine (2023March#01 and 2023March#02) Research Ethics Committees.

## 3. Results

### 3.1. General Description of the Sample

**Figure 1:**
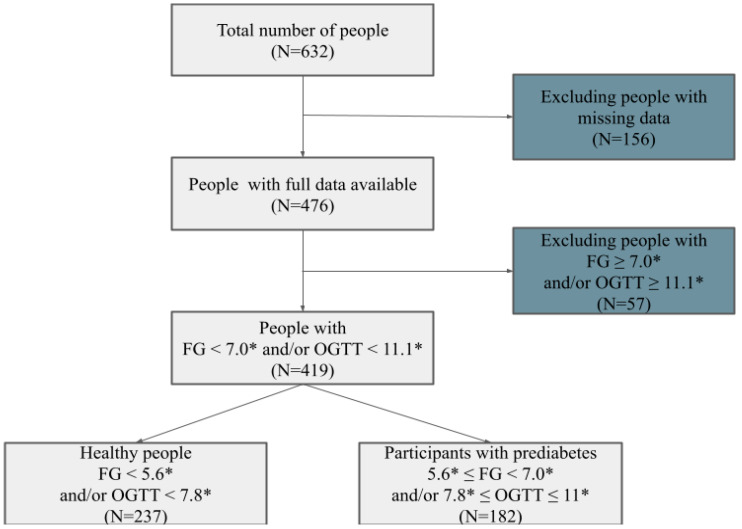
Flow chart for the participant selection for the study. Notes: FG—fasting glucose, OGTT—oral glucose tolerance test; *—mmol/L.

Initially, the dataset contained records of 632 participants initially recruited in 2012–24. A total of 213 respondents were excluded due to missing data and FG ≥ 7.0 mmol/L or OGTT ≥ 11.1 mmol/L indicators, while fasting glucose and OGTTs were only available for 476 individuals who had fully completed data. The total sample comprised 419 subjects, of which 182 (43.4% of the study population) were compatible with PreDM criteria (Figure 1).

The general characteristics of the study population are shown in Table 1. The PreDM group showed a higher prevalence of obesity, high BMI, and age of 50 and older. No differences in the proportions of men and women were identified. The PreDM showed a higher proportion of older participants (*p* < 0.0001).

The proportion of ethnic Kazakhs did not show significant differences between both groups. Table 2 reports the biochemical, clinical, and metabolic characteristics of the study group. The median fasting (6.2 (0.7) vs. 5.0 (0.54) mmol/L, *p* < 0.001) and 2 h (5.8 (1.85) vs. 5.3 (0.8) mmol/L, *p* < 0.001) plasma glucose levels after oral glucose challenge in the PreDM group were significantly higher than those of healthy individuals.

Regarding the study’s power to detect significant statistical differences, for α = 0.05 and an effect size (d) of 0.5, the power to detect significant differences in the prevalence of PreDM is approximately 99%. The study had a statistical power of 99.6% to detect the observed differences in fasting glucose levels across the four latent classes (Cohen’s f = 0.42).

### 3.2. LCA Group Definitions and Their Cardio-Metabolic Characteristics

- Class 1: Healthy, low risk (*n* = 62; 34.1% of PreDM group; median age: 35 years, BMI: 24.8 kg/m^2^), predominantly IFG (95%, 95% CI: 89–98%).
- Class 2: Overweight, moderate risk (*n* = 48; 26.4%; median age: 42 years, BMI: 27.5 kg/m^2^), with 18% IFG + IGT (95% CI: 10–29%).
- Class 3: Older, overweight, high risk (*n* = 42; 23.1%; median age: 55 years, BMI: 29.2 kg/m^2^), with 33% IFG + IGT (95% CI: 21–47%) and 40% β-cell dysfunction (HOMA-β ≤ 50, 95% CI: 27–55%).
- Class 4: Obese, middle-aged to older, very high risk (*n* = 30; 16.5%; median age: 52 years, BMI: 32.1 kg/m^2^), with 30% IFG + IGT (95% CI: 17–47%) and 50% β-cell dysfunction (95% CI: 34–66%).

Significant differences were found in FG (*p* < 0.001), OGTT (*p* = 0.003), and HOMA-β (*p* = 0.006) across classes, but not HOMA-IR (*p* = 0.12). Bonferroni post hoc tests confirmed FG differences between Class 1 vs. Classes 3–4 (*p* < 0.01). The cardiovascular risk profiles of the four LCAs are shown in Section 3.2 and their glucose metabolism characteristics in Table 3. Table 4 shows AIC (4235.6) and BIC (4356.2) favored a four-class model.

The violin plots in Figure S1 in the Supplementary Material display the distribution of anthropometric and biochemical markers across the four latent classes. Body Mass Index, waist circumference, and hip circumference progressively increase from the young mid-risk to the obese high-risk group, reflecting the severity of adiposity. The obese high-risk group shows the highest median and widest spread, indicating a broad range of obesity-related risk. Elevated blood pressure is most prominent in the older overweight high-risk and obese high-risk groups, suggesting a clustering of hypertension with metabolic dysfunction. Triglycerides are substantially higher in the obese high-risk group, consistent with IR. HDL cholesterol is lowest in the same group, reinforcing the adverse lipid pattern. LDL and total cholesterol show less marked variation, though slightly higher medians are noted in the higher-risk groups.

Table 4 shows the AIC and BIC of the different groups tested while conducting the LCA, being the most favorable when selecting four subgroups.

## 4. Discussion

This study highlights a high prevalence of PreDM (43.4%) in a working-age Kazakh population and reveals substantial heterogeneity in its presentation. This percentage is much higher than previously reported rates, likely due to under-diagnosis, less representative study populations, and limited screening methods in earlier studies. Four distinct latent classes were identified, reflecting differences in age, anthropometric measurements, cardiovascular risk factors, and glucose metabolism profiles.

The overall high prevalence of PreDM in this population is consistent with regional data, though higher than previous estimates in Kazakhstan, most likely reflecting differences in the populations analyzed. The PreDM population has a significantly higher prevalence of obesity (46.7% vs. 27.4%, *p* < 0.000) and abdominal obesity (67.0% vs. 41.7%, *p* < 0.000) compared to the healthy population (Table 1) [19,20], and is more frequent at advanced ages [21,22]. Patients with PreDM already show some vascular complications typically associated with DM [23] and associations with abnormal irregular fluctuations in blood pressure [24] and elevated cardiovascular risk [25,26].

The second finding of this work is the significant heterogeneity in the PreDM population. Previous LCA and cluster analysis studies also identified heterogeneous risk profiles within PreDM, confirming that PreDM encompasses subtypes with variable pathophysiological features, ranging from isolated IFG to combined IFG/IGT with β-cell dysfunction. Our results add to this evidence from a Central Asian context, suggesting that population-specific characteristics such as age distribution and ethnic composition shape the distribution of metabolic phenotypes.

LCA effectively uncovers subclinical heterogeneity in a group that might all meet generic “PreDM” criteria but differ in underlying mechanisms (e.g., IR vs. β-cell failure). These differences may be associated with variability in the future risk of progression to T2DM or cardiovascular disease, as well as a potential for differential response to preventive interventions. Possible pathophysiological differences in the four groups may be as follows:

- Age-Related Changes in Glucose Metabolism: Older age in Clusters 3 and 4 is associated with a combined increase in IR and progressive β-cell decline, while higher IFG but lower IGT in younger participants (Cluster 1), may reflect hepatic IR and still-compensating β-cells.
- Obesity and IR Patterns: The worst glycemia is identified in Cluster 4, characterized by high-risk obesity, but with no significant difference in HOMA-IR. This may reflect adipose-related IR affecting both the liver and peripheral tissues, and failing compensatory hyperinsulinemia due to β-cell exhaustion. Despite the lack of statistically significant differences in HOMA-IR between clusters, the observed decline in β-cell function in this cluster suggests that depletion of β-cell insulin secretory capacity, rather than insulin resistance itself, may play a major role in diabetes progression in this population.
- β-cell function and compensation: The declining HOMA-β across clusters indicates progressive β-cell dysfunction, with the lowest values observed in Clusters 3 and 4. Despite similar HOMA-IR values across groups, this suggests insufficient compensation in older and obese groups.
- Glucose dysregulation: suggested by diverse IFG vs. IGT vs. IFG + IGT profiles:
  1. High IFG: predominantly hepatic IR (Cluster 1);
  2. IFG + IGT: mixed and advanced hepatic and muscle IR (in Clusters 3 and 4);
  3. Increasing IGT/IFG + IGT: a transitional group with early decline in β-cell function (Cluster 2).

The glucose metabolism disturbance leading to PreDM and eventually to T2DM and further complications varies across individuals, depending on different risk factors. This heterogeneity makes preventing T2DM difficult [27,28,29,30]. People with PreDM also differ in their characteristics and in how they respond to prevention strategies [31,32,33]. Studies show that 36–60% of individuals with PreDM can return to normal blood sugar levels [34,35], suggesting that environmental, genetic, and ethnic factors influence both the risk of T2DM and the effectiveness of prevention efforts [36,37].

Unsupervised learning methods, such as cluster analysis or LCA, have been used to generate homogeneous groups of PreDM that may reflect sub-phenotypes expressing different pathophysiological trajectories, risk of progression and preventive approaches. Wagner identified six distinct clusters: while three sub-phenotypes had increased glycemia, only individuals in Clusters 5 and 3 had short-term T2DM risk. By contrast, those in Cluster 6 had a moderate risk of T2DM, but an increased risk of kidney disease and all-cause mortality [38]. Prystupa found six clusters with different risks of developing T2DM and overall mortality [39]. Cho found six population clusters with significantly different prevalence rates of T2DM which also showed different clinical and biochemical profiles [40]. Yacaman Mendez identified six risk phenotypes: very-low-risk (VLR), low-risk low-β-cell-function (LRLB), low-risk high-β-cell-function (LRHB), high-risk high-blood-pressure (HRHBP), high-risk β-cell-failure (HRBF), and high-risk insulin-resistant (HRIR). The HRHBP, HRBF, and HRIR clusters showed a higher risk of developing T2DM [41]. Li, using K-means clustering, obtained six clusters of individuals presenting disparate patterns of polygenic risk scores and different patterns of metabolic traits [42]. Two potential genetic subtypes of PreDM showed relatively high risk of T2DM over time, observing also that individuals in one subtype may realize extra benefits in terms of risk reduction from a healthy lifestyle.

Based on biomarkers of subclinical inflammation, Huemer derived an inflammation-related score (“inflammatory load”) using principal component analysis, identifying that high cardio-metabolic risk corresponded to the high inflammatory load in some clusters, but not to the lower inflammatory load of high-risk clusters [43].

Several authors have also explored how ethnic and genetic differences may be associated with differences in PreDM characteristics. Fowler described that impaired β-cell function may underlie T2DM etiology more profoundly in Non-Hispanic Blacks. In contrast, hepatic dysfunction, lipid metabolism abnormalities, and genetic IR contribute to T2DM etiology to a greater degree in both Non-Hispanic Blacks and Hispanics [44]. Analyzing data from Taiwan and UK biobanks, Onthoni identified two stable clusters that represent high- and low-risk diabetes groups in both biobanks. The high-risk clusters showed higher diabetes incidence, with 15.7% in Taiwan and 13.0% in the UK, compared to 7.3% and 9.1% in the low-risk clusters, respectively. In Taiwan, the high-risk group also exhibited significantly higher BMI, fasting glucose, and triglycerides, while in the UK there was marginal significance in BMI and other metabolic indicators [45].

LCA is a robust statistical approach that, applying mixture modeling, permits us to identify best-fitting optimal aggregation of cases based on the existence of unobserved latent classes or subgroups within the data classes [46]. Unlike traditional analyses, which seek to elucidate associations between predefined independent variables and known outcomes, LCA delineates homogeneous groups of individuals based on shared patterns across multiple baseline variables. While sharing conceptual similarities with cluster analysis, LCA is grounded in a measurement model akin to factor analysis, facilitating the detection of inherent heterogeneity in population-level individual variations that may not be directly observable [47].

The identified LCA could possibly offer a framework for precision medicine in PreDM management. For instance, Class 1 with low risk may be effectively managed with lifestyle modification alone. Class 2 represents an intermediate group suitable for targeted behavioral counseling. Classes 3 and 4 are more likely to warrant early pharmacological interventions.

This study has also limitations. Firstly, this is a small-sample-size study from a selected working-age population. Further validation is required in larger and more diverse samples to confirm if the results could be generalized. Additionally, the glucose homeostasis indices may be valid only for the specific Kazakh population in which they were obtained as they may be influenced by ethnic or genetic factors. Secondly, the cross-sectional design of the study precludes inference of causality or progression to T2DM. This study relies solely on WHO criteria and in those in the sample had these data available (IFG: 6.1–6.9 mmol/L; IGT: 7.8–11.1 mmol/L via OGTT); HbA1c was omitted, limiting comparability to global studies utilizing the metrics of the American Diabetes Association [2]. In this study, we established specific cut-off points for HOMA-IR and HOMA-β; other cut-off points may have rendered different results. The inclusion of inflammatory markers, such as C-reactive protein, and liver enzymes were not collected in this study so although they may have meaningful associations with glucose dysregulation, their effect was not possible to estimate. Additionally, lifestyle, dietary, and genetic data were also unavailable, which may have further informed class differentiation. A lack of standardized universal insulin assays limits their use for routine assessment of IR in the clinical setting and may have affected our results. Lastly, the aim of this study was not to elucidate the mechanistic explanations of the associations that may have been identified by analyzing these data. An important rationale for our approach is that OGTT and insulin measures are not routinely available in general practice settings, particularly in low- and middle-income countries. By building the LCA model on simple cardiovascular risk factors, we sought to explore whether readily obtainable clinical and biochemical data could still capture meaningful heterogeneity among individuals with PreDM, making these findings potentially more translatable to clinical settings where physicians must often make preventive decisions without detailed metabolic testing. Nevertheless, it should be emphasized that these subgroups are not mechanistic categories, and their predictive value for progression to T2DM or cardiovascular outcomes requires validation in longitudinal studies. Future research should also compare the performance of LCA-based subgrouping against existing simple risk scores (e.g., FINDRISC, ADA risk score) to assess incremental clinical value.

Despite these limitations, this study is one of the first that has applied data-driven strategies to stratify PreDM in Central Asia, confirming that LCA is an innovative and effective method for grouping individuals with PreDM. It has the advantage, compared to hierarchical or k-means clustering, of the possibility of statistics (AIC, BIC) tests that help to determine the best number of classes. The sample size was powered enough to identify the prevalence of PreDM and to detect clinically meaningful variations in glucose metabolism profiles.

## 5. Conclusions

The application of LCA showed the heterogeneity that exists in the widespread PreDM population in Kazakhstan. Four classes emerged, characterized by different cardio-metabolic profiles, suggesting possible different physio-pathological pathways and that different interventions may be appropriate to prevent the onset of T2DM in each of them [48,49]. Furthermore, the identified profiles can be leveraged to facilitate precision management strategies, underscoring the imperative for their implementation [50]. The four LCA-derived groups align well with clusters identified in other populations, although in this study β-cell deficit is a key differentiator, especially in older, overweight high-risk individuals. However, non-significant HOMA-IR differences and lack of lipid/inflammation data set this study apart, suggesting unique cohort characteristics or methodological influences.

Further longitudinal studies are needed to investigate the incidence of Type 2 Diabetes, but this study highlights the importance of determining patients’ cardio-metabolic profile for effective T2DM prevention, as well as their ethnic background. These pathophysiological differences should determine the appropriate therapeutic approach [51,52,53,54,55]. Kazakhstan is an ethnically diverse Central Asian country whose genetic characteristics may hold an intermediate position between South and Eastern Asian and European populations [55]. Evidence-based preventive interventions will require contextualization based on the characteristics of the populations that will receive them [56,57]. Diabetes is a complex disease with a complex interplay of genetic, clinical, and environmental factors, and its pathophysiology may vary substantially across populations. Therefore, the specific subgroups identified in one population, like those found in this study, should not be expected to be replicated with the same profiles in other settings. Instead, heterogeneity across populations should be anticipated and understood as a reflection of contextual influences on risk of progression to T2DM and, later, to diabetes-related complications [58]. From this perspective, the possible lower generalizability of context-specific studies may not be less problematic, as the aim and contribution of each study is to mapping the diversity of PreDM phenotypes in specific populations, rather than aiming for universal generalizability.

## Figures and Tables

**Table 1:** General characteristics of the study population.

Characteristics			Normoglycemic		Prediabetes		*p*
			Frequency	%	Frequency	%	
Sex	Men		59	24.9	59	32.4	NS
	Women		178	75.1	123	67.6	
Age groups (years)	20–29		8	3.40	0	0	<0.000
	30–39		75	31.6	21	11.5	
	40–49		66	27.8	38	20.9	
	50–59		61	25.7	62	34.1	
	60–69		27	11.4	61	33.5	
Kazakh			204	86.1	166	91.2	NS
BMI (kg/m^2^)		Normal	97	41.0	27	14.8	<0.000
		Overweight	75	31.6	70	38.5	
		Obese	65	27.4	85	46.7	
Waist circumference (cm)		M: <94; F: <80	91	38.4	27	14.8	<0.000
		M: 94–102; F: 80–88	47	19.8	33	18.1	
		M: 102<; F: 88<	99	41.7	122	67.0	
Insulin resistance			38	16.0	58	31.9	<0.000
Poor beta-cell function			22	9.28	87	47.8	<0.000
Total number of participants			237	56.6	182	43.4	

**Table 2:** Clinical, biochemical, and metabolic characteristics of the study population.

Characteristics	Normoglycemic		Prediabetes		*p*
	Median	IQR	Median	IQR	
Age (years)	45	14	55	16	<0.000
BMI	26.30	7.89	29.38	7.30	<0.000
Waist circumference (cm)	89	20	97	15	<0.000
Hip circumference (cm)	101	14	108	13	<0.000
SBP (mmHg)	110	30	140	40	<0.000
DBP (mmHg)	80	20	82.5	10	<0.000
Total cholesterol (mmol/L)	4.80	0.8	5.10	1.10	<0.000
LDL-cholesterol (mmol/L)	2.10	0.71	2.36	0.69	<0.000
HDL-cholesterol (mmol/L)	1.26	0.24	1.17	0.25	0.009
TG (mmol/L)	1.97	1.21	2.07	0.92	0.034
Fasting glucose (mmol/L)	5.0	0.54	6.20	0.7	<0.000
OGTT (mmol/L)	5.3	0.8	5.80	1.85	<0.000
Fasting insulin (µU/mL)	7.73	4.77	7.52	5.02	<0.000
HOMA-IR	1.67	1.02	2.02	1.36	<0.000
HOMA-beta	114.0	92.99	52.84	45.69	<0.000

Notes: SBP—systolic blood pressure, DBP—diastolic blood pressure, LDL—low-density lipoprotein, HDL—high-density lipoprotein, TG—triglycerides, OGTT—oral glucose tolerance test.

**Table 3:** Cardiovascular risk factors and glucose metabolism indexes of the 4 prediabetes latent classes.

	Healthy, Low Risk	Overweight, Moderate Risk	Older, Overweight, High Risk	Obese, Middle-Aged to Older, Very High Risk	*p*
%	20.8	19.8	38.5	20.8	
Fasting glucose	5.99	6.17	6.28	6.68	0.001
OGTT	5.51	6.19	6.51	6.58	0.003
HOMA IR	2.35	2.25	1.96	2.34	0.194
HOMA beta	72.21	65.63	52.75	51.83	0.006
IFG	95.0%	78.9%	68.9%	62.5%	0.001
IGT	0%	2.6%	5.4%	5.0%	0.030
IFG + IGT	5.0%	18.4%	25.7%	32.5%	0.001
IR	45.0%	36.8%	23.0%	35.0%	0.097
Beta-cell deficit	30.0%	39.5%	60.8%	55.0%	0.008

Notes: OGTT—oral glucose tolerance test, IR—insulin resistance, IFG—impaired fasting glucose, IGT—impaired glucose tolerance.

**Table 4:** Aikake (AIC) and Bayesian Information Criteria (BIC) values for the different Latent Class Analysis.

Model	Log-likelihood	df	AIC	BIC
2 Classes	−4870.519	34	9809.039	9919.794
3 Classes	−4792.777	46	9677.554	9827.399
4 Classes	−4741.992	58	9599.985	9788.92

## Data Availability

The data presented in this study are available upon request from the corresponding author (Because of confidentiality and approval from ethics committees, data is available on request to the corresponding author).

## Supplementary Materials

The following supporting information can be downloaded at: https://www.mdpi.com/article/10.3390/medsci13040243/s1, Figure S1. Violin plots representing the distribution of participants included in the 4 LCA groups identified.

## Abbreviations

The following abbreviations are used in this manuscript:

T2DM	Type 2 Diabetes Mellitus
PreDM	Prediabetes
LCA	Latent Class Analysis
BMI	Body Mass Index
HOMA	Homeostatic Model Assessment
OGTT	Oral Glucose Tolerance Test
IR	Insulin Resistance
IFG	Impaired Fasting Glucose
IGT	Impaired Glucose Tolerance

## References

[1] The Lancet Diabetes & Endocrinology (2025). Prediabetes: Much More Than Just a Risk Factor. Lancet Diabetes Endocrinol..

[2] American Diabetes Association (2024). Diabetes: Standards of Care in Diabetes—2024. Diabetes Care.

[3] Richter B., Hemmingsen B., Metzendorf M.I., Takwoingi Y. (2018). Development of type 2 diabetes mellitus in people with intermediate hyperglycaemia. Cochrane Database Syst. Rev..

[4] Rooney M.R., He J.H., Salpea P., Genitsaridi I., Magliano D.J., Boyko E.J., Wallace A.S., Fang M., Selvin E. (2025). Global and regional prediabetes prevalence: Updates for 2024 and projections for 2050. Diabetes Care.

[5] Diabetes Prevention Program Research Group (2002). Reduction in the Incidence of Type 2 Diabetes with Lifestyle Intervention or Metformin. N. Engl. J. Med..

[6] Hauguel-Moreau M., Hergault H., Cazabat L., Pepin M., Beauchet A., Aidan V., Ouadahi M., Josseran L., Hage M., Rodon C. (2023). Prevalence of prediabetes and undiagnosed diabetes in a large urban middle-aged population: The CARVAR 92 cohort. Cardiovasc. Diabetol..

[7] Orazumbekova B., Issanov A., Atageldiyeva K., Berkinbayev S., Junusbekova G., Danyarova L., Shyman Z., Tashmanova A., Sarria-Santamera A. (2022). Prevalence of Impaired Fasting Glucose and Type 2 Diabetes in Kazakhstan: Findings from Large Study. Front. Public Health.

[8] Aleman-Vega G., Garrido-Elustondo S., Del Cura-Gonzalez I., Sarria-Santamera A. (2017). Is a maintained glycemia between 110/125 mg/dl a risk factor in the development of diabetes?. Aten. Primaria.

[9] Davidson K.W., Barry M.J., Mangione C.M., Cabana M., Caughey A.B., Davis E.M., Donahue K.E., Doubeni C.A., Krist A.H., Kubik M. (2021). US Preventive Services Task Force Screening for Prediabetes Type 2 Diabetes: US Preventive Services Task Force Recommendation Statement. JAMA.

[10] Hollander P., Spellman C. (2012). Controversies in prediabetes: Do we have a diagnosis?. Postgrad. Med..

[11] Barbu E., Popescu M.R., Popescu A.C., Balanescu S.M. (2021). Phenotyping the prediabetic population-a closer look at intermediate glucose status and cardiovascular disease. Int. J. Mol. Sci..

[12] Tabák A.G., Herder C., Rathmann W., Brunner E.J., Kivimäki M. (2012). Prediabetes: A high-risk state for diabetes development. Lancet.

[13] Barthow C., Pullon S., McKinlay E., Krebs J. (2022). It is time for a more targeted approach to prediabetes in primary care in Aotearoa New Zealand. J. Prim. Health Care.

[14] Von Elm E., Altman D.G., Egger M., Pocock S.J., Gøtzsche P.C., Vandenbroucke J.P., STROBE Initiative (2007). The Strengthening the Reporting of Observational Studies in Epidemiology (STROBE) statement: Guidelines for reporting observational studies. Lancet.

[15] Geloneze B., Vasques A.C.J., Stabe C.F.C., Pareja J.C., Rosado L.E.F.P., Queiroz E.C., Tambascia M.A. (2009). HOMA1-IR and HOMA2-IR indexes in identifying insulin resistance and metabolic syndrome—Brazilian Metabolic Syndrome Study (BRAMS). Arq. Bras. Endocrinol. Metabol..

[16] Kim B., Choi H.Y., Kim W., Ahn C., Lee J., Kim J.G., Kim J., Shin H., Yu J.M., Moon S. (2018). The cut-off values of surrogate measures for insulin resistance in the Korean population according to the Korean Genome and Epidemiology Study (KOGES). PLoS ONE.

[17] Basukala P., Jha B., Yadav B.K., Shrestha P.K. (2018). Determination of insulin resistance and Beta-Cell function using homeostatic model assessment in Type 2 diabetic patients at diagnosis. J. Diabetes Metab..

[18] Lanza S.T., Rhoades B.L. (2013). Latent class analysis: An alternative perspective on subgroup analysis in prevention and treatment. Prev. Sci..

[19] Akter S., Rahman M.M., Abe S.K., Sultana P. (2014). Prevalence of diabetes and prediabetes and their risk factors among Bangladeshi adults: A nationwide survey. Bull. World Health Organ..

[20] Satman I., Omer B., Tutuncu Y., Kalaca S., Gedik S., Dinccag N., Karsidag K., Genc S., Telci A., Canbaz B. (2013). Twelve-year trends in the prevalence and risk factors of diabetes and prediabetes in Turkish adults. Eur. J. Epidemiol..

[21] Bocquet V., Ruiz-Castell M., de Beaufort C., Barré J., de Rekeneire N., Michel G., Donahue R.P., Kuemmerle A., Stranges S. (2019). Public health burden of pre-diabetes and diabetes in Luxembourg: Finding from the 2013–2015 European Health Examination Survey. BMJ Open.

[22] Vera-Ponce V.J., Zuzunaga-Montoya F.E., Vásquez-Romero L.E.M., Loayza-Castro J.A., Paucar C.R.I., De Carrillo C.I.G., Valladares-Garrido M.J., Medina M.P. (2024). Anthropometric measures of obesity as risk indicators for prediabetes. A systematic review and meta-analysis. Diabetes Epidemiol. Manag..

[23] Beulens J.W.J., Rutters F., Ryden L., Schnell O., Mellbin L., Hart H.E., Vos R.C. (2019). Risk and management of pre-diabetes. Eur. J. Prev. Cardiol..

[24] Gupta A.K., Greenway F.L., Cornelissen G., Pan W., Halberg F. (2008). Prediabetes is associated with abnormal circadian blood pressure variability. J. Hum. Hypertens..

[25] Nanri A., Nakagawa T., Kuwahara K., Yamamoto S., Honda T., Okazaki H., Uehara A., Yamamoto M., Miyamoto T., Kochi T. (2015). Development of Risk Score for Predicting 3-Year Incidence of Type 2 Diabetes: Japan Epidemiology Collaboration on Occupational Health Study. PLoS ONE.

[26] Cai X., Zhang Y., Li M., Wu J.H., Mai L., Li J., Yang Y., Hu Y., Huang Y. (2020). Association between prediabetes and risk of all cause mortality and cardiovascular disease: Updated meta-analysis. BMJ.

[27] Armato J.P., DeFronzo R.A., Abdul-Ghani M., Ruby R.J. (2018). Successful treatment of prediabetes in clinical practice using physiological assessment (STOP DIABETES). Lancet Diabetes Endocrinol..

[28] Hjellvik V., Strøm H., Sakshaug S. (2012). Body mass index, triglycerides, glucose, and blood pressure as predictors of type 2 diabetes in a middle-aged Norwegian cohort of men and women. Clin. Epidemiol..

[29] Bellou V., Belbasis L., Tzoulaki I., Evangelou E. (2018). Risk factors for type 2 diabetes mellitus: An exposure-wide umbrella review of meta-analyses. PLoS ONE.

[30] Glechner A., Keuchel L., Affengruber L., Titscher V., Sommer I., Matyas N., Wagner G., Kien C., Klerings I., Gartlehner G. (2018). Effects of lifestyle changes on adults with prediabetes: A systematic review and meta-analysis. Prim. Care Diabetes.

[31] Zhang Y., Pan X.-F., Chen J., Xia L., Cao A., Zhang Y., Wang J., Li H., Yang K., Guo K. (2020). Combined lifestyle factors and risk of incident type 2 diabetes and prognosis among individuals with type 2 diabetes: A systematic review and meta-analysis of prospective cohort studies. Diabetologia.

[32] Luo Y., Wang H., Zhou X., Chang C., Chen W., Guo X., Yang J., Ji L., Paul S.K. (2022). A randomized controlled clinical trial of lifestyle intervention and pioglitazone for normalization of glucose status in Chinese with prediabetes. J. Diabetes Res..

[33] Kim C.H., Kim H.K., Kim E.H., Bae S.J., Choe J., Park J.Y. (2016). Risk of progression to diabetes from prediabetes defined by HbA1c or fasting plasma glucose criteria in Koreans. Diabetes Res. Clin. Pract..

[34] Bennasar-Veny M., Fresneda S., López-González A., Busquets-Cortés C., Aguiló A., Yañez A.M. (2020). Lifestyle and progression to type 2 diabetes in a cohort of workers with prediabetes. Nutrients.

[35] Giráldez-García C., Cea-Soriano L., Albaladejo R., Franch-Nadal J., Mata-Cases M., Díez-Espino J., Artola S., Serrano R., Regidor E., PREDAPS Study Group (2021). The heterogeneity of reversion to normoglycemia according to prediabetes type is not explained by lifestyle factors. Sci. Rep..

[36] Sentell T.L., He G., Gregg E.W., Schillinger D. (2012). Racial/ethnic variation in prevalence estimates for United States prediabetes under alternative 2010 American Diabetes Association criteria: 1988–2008. Ethn. Dis..

[37] Vicks W.S., Lo J.C., Guo L., Rana J.S., Zhang S., Ramalingam N.D., Gordon N.P. (2022). Prevalence of prediabetes and diabetes vary by ethnicity among U.S. Asian adults at healthy weight, overweight, and obesity ranges: An electronic health record study. BMC Public Health.

[38] Wagner R., Heni M., Tabák A.G., Machann J., Schick F., Randrianarisoa E., de Angelis M.H., Birkenfeld A.L., Stefan N., Peter A. (2021). Pathophysiology-based subphenotyping of individuals at elevated risk for type 2 diabetes. Nat. Med..

[39] Prystupa K., Delgado G.E., Moissl A.P., Kleber M.E., Birkenfeld A.L., Heni M., Fritsche A., März W., Wagner R. (2023). Clusters of prediabetes and type 2 diabetes stratify all-cause mortality in a cohort of participants undergoing invasive coronary diagnostics. Cardiovasc. Diabetol..

[40] Cho S.B., Kim S.C., Chung M.G. (2019). Identification of novel population clusters with different susceptibilities to type 2 diabetes and their impact on the prediction of diabetes. Sci. Rep..

[41] Méndez D.Y., Zhou M., Lagerros Y.T., Velasco D.V.G., Tynelius P., Gudjonsdottir H., de Leon A.P., Eeg-Olofsson K., Östenson C.-G., Brynedal B. (2022). Characterization of data-driven clusters in diabetes-free adults and their utility for risk stratification of type 2 diabetes. BMC Med..

[42] Li Y., Chen G.C., Moon J.Y., Arthur R., Sotres-Alvarez D., Daviglus M.L., Pirzada A., Mattei J., Perreira K.M., Rotter J.I. (2024). Genetic Subtypes of Prediabetes, Healthy Lifestyle, and Risk of Type 2 Diabetes. Diabetes.

[43] Huemer M.-T., Spagnuolo M.C., Maalmi H., Wagner R., Bönhof G.J., Heier M., Koenig W., Rathmann W., Prystupa K., Nano J. (2025). Phenotype-based clusters, inflammation and cardiometabolic complications in older people before the diagnosis of type 2 diabetes: KORA F4/FF4 cohort study. Cardiovasc. Diabetol..

[44] Fowler L.A., Fernández J.R., O’Neil P.M., Parcha V., Arora P., Shetty N.S., Cardel M.I., Foster G.D., Gower B.A. (2025). Genetic Risk Phenotypes for Type 2 Diabetes Differ with Ancestry in US Adults with Diabetes and Overweight/Obesity. Arch. Med. Res..

[45] Onthoni D.D., Chen Y.E., Lai Y.H., Li G.H., Zhuang Y.S., Lin H.M., Hsiao Y.P., Onthoni A.I., Chiou H.Y., Chung R.H. (2025). Clustering-based risk stratification of prediabetes populations: Insights from the Taiwan and UK Biobanks. J. Diabetes Investig..

[46] Calfee C.S., Delucchi K.L., Sinha P., Matthay M.A., Hackett J., Shankar-Hari M., McDowell C., Laffey J.G., O’Kane C.M., McAuley D.F. (2018). Acute respiratory distress syndrome subphenotypes and differential response to simvastatin: Secondary analysis of a randomised controlled trial. Lancet Respir. Med..

[47] Wu Y., Hu H., Cai J., Chen R., Zuo X., Cheng H., Yan D. (2021). Applying latent class analysis to risk stratification of incident diabetes among Chinese adults. Diabetes Res. Clin. Pract..

[48] Lanza S.T., Rhoades B.L., Nix R.L., Greenberg M.T. (2010). Modeling the interplay of multilevel risk factors for future academic and behavior problems: A person-centered approach. Dev. Psychopathol..

[49] Færch K., Borch-Johnsen K., Holst J.J., Vaag A. (2009). Pathophysiology and etiology of impaired fasting glycaemia and impaired glucose tolerance: Does it matter for prevention and treatment of type 2 diabetes. Diabetologia.

[50] Franks P.W., Sargent J.L. (2024). Diabetes and obesity: Leveraging heterogeneity for precision medicine. Eur. Heart J..

[51] Nathan D.M., Davidson M.B., DeFronzo R.A., Heine R.J., Henry R.R., Pratley R., Zinman B. (2007). Impaired Fasting Glucose and Impaired Glucose Tolerance: Implications for care. Diabetes Care.

[52] Faerch K., Vaag A., Holst J.J., Hansen T., Jorgensen T., Borch-Johnsen K. (2009). Natural history of insulin sensitivity and insulin secretion in the progression from normal glucose tolerance to impaired fasting glycemia and impaired glucose tolerance-the Inter99 study. Diabetes Care.

[53] Tuomilehto J., Lindström J., Eriksson J.G., Valle T.T., Hämäläinen H., Ilanne-Parikka P., Keinänen-Kiukaanniemi S., Laakso M., Louheranta A., Rastas M. (2001). Prevention of type 2 diabetes mellitus by changes in lifestyle among subjects with impaired glucose tolerance. N. Engl. J. Med..

[54] Kodama K., Tojjar D., Yamada S., Toda K., Patel C.J., Butte A.J. (2013). Ethnic differences in the relationship between insulin sensitivity and insulin response: A systematic review and meta-analysis. Diabetes Care.

[55] Yabe D., Seino Y., Fukushima M., Seino S. (2015). β cell dysfunction versus insulin resistance in the pathogenesis of type 2 diabetes in East Asians. Curr. Diabetes Rev..

[56] Sikhayeva N., Talzhanov Y., Iskakova A., Dzharmukhanov J., Nugmanova R., Zholdybaeva E., Ramanculov E. (2018). Type 2 diabetes mellitus: Distribution of genetic markers in Kazakh population. Clin. Interv. Aging.

[57] Saruarov Y., Nuskabayeva G., Gencer M.Z., Sadykova K., Zhunissova M., Tatykayeva U., Iskandirova E., Sarsenova G., Durmanova A., Gaipov A. (2023). Associations of Clusters of Cardiovascular Risk Factors with Insulin Resistance and ?-Cell Functioning in a Working-Age Diabetic-Free Population in Kazakhstan. Int. J. Environ. Res. Public Health.

[58] Misra S., Aguilar-Salinas C.A., Chikowore T., Konradsen F., Ma R.C., Mbau L., Mohan V., Morton R.W., Nyirenda M.J., Tapela N. (2023). The case for precision medicine in the prevention, diagnosis, and treatment of cardiometabolic diseases in low-income and middle-income countries. Lancet Diabetes Endocrinol..

