# Supplementary material for: Identifying Cardio-Metabolic Subtypes of Prediabetes Using Latent Class Analysis
Source: Med Sci (Basel). 2025 Oct 25;13(4):243. doi: 10.3390/medsci13040243 (PMC12641829; doi:10.3390/medsci13040243)
Supplement: Supplementary file 1 [file medsci-13-00243-s001.zip › medsci-3893782-supplementary.pdf]

Figure S1. Violin plots representing the distribution of participants included in the 4 LCA groups identified.

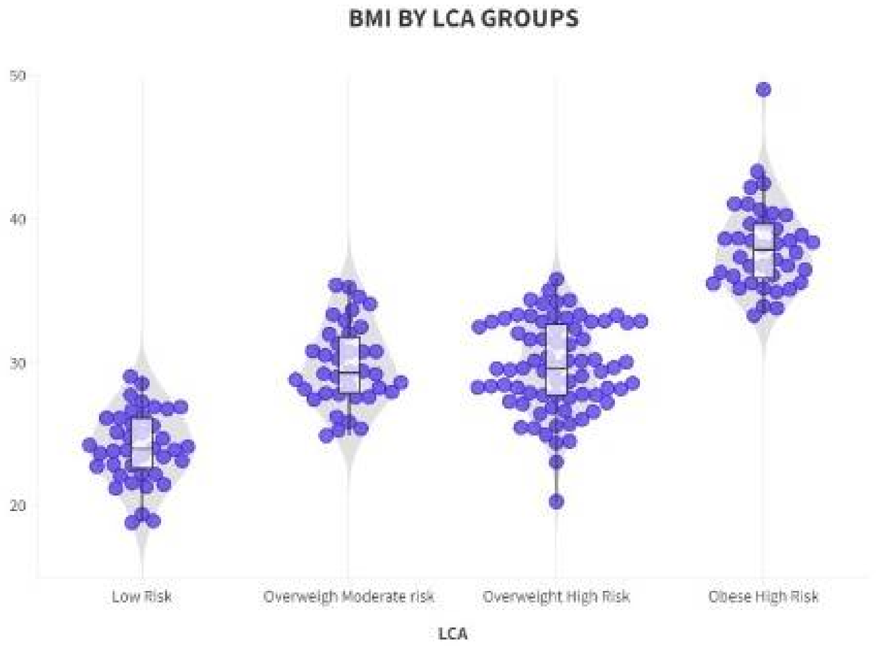

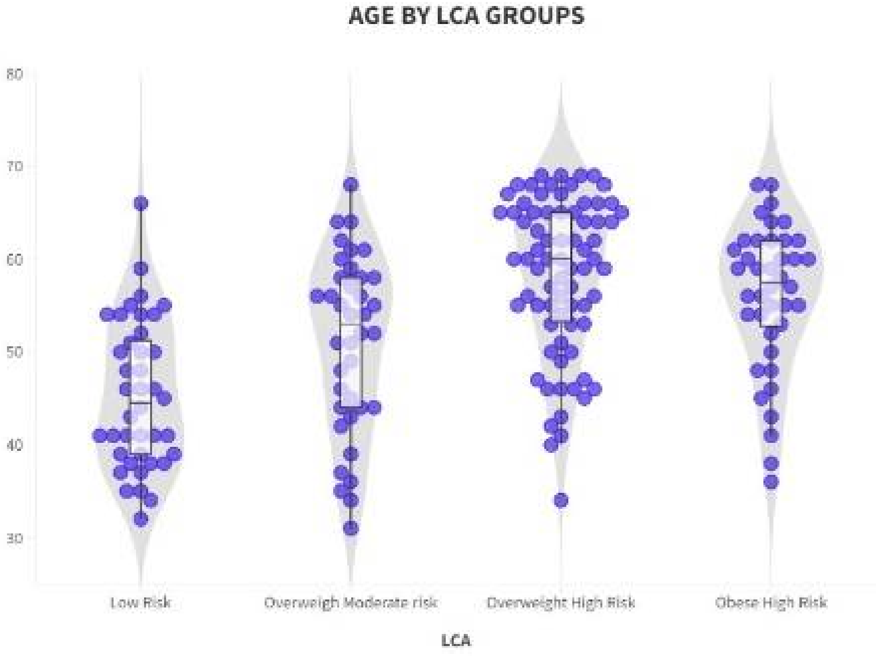

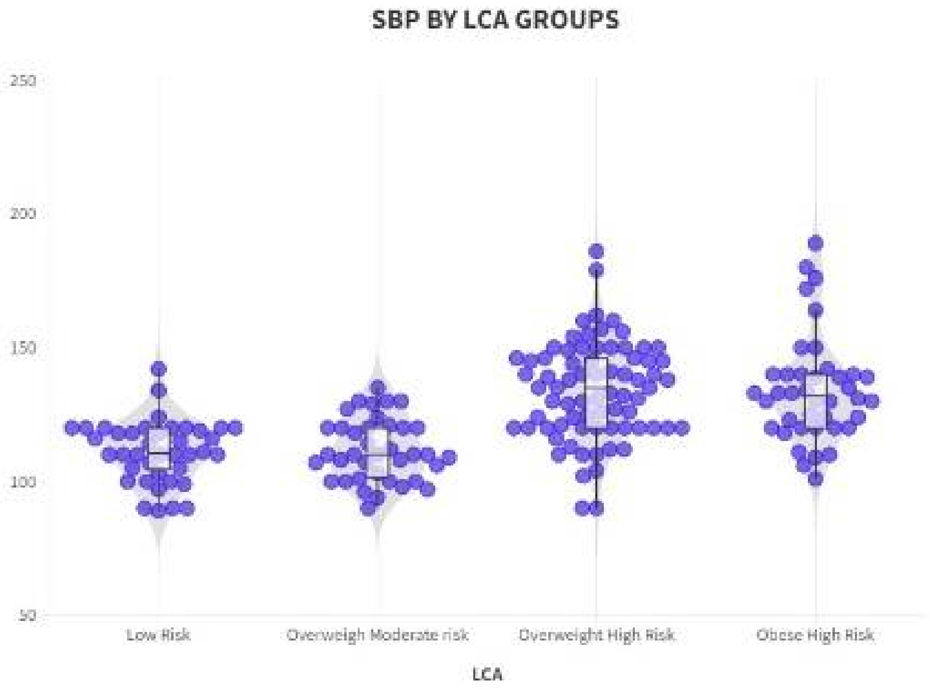

SBP BY LCA GROUPS
250
200
150
100
50
Low Risk
Overweigh Moderate risk
Overweight High Risk
Obese High Risk
LCA

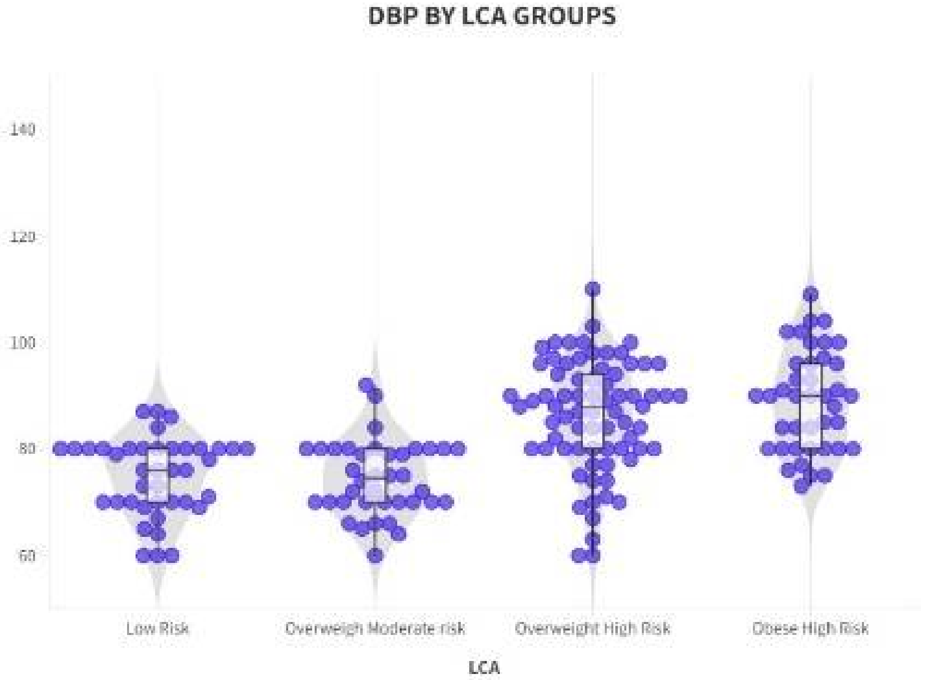

DBP BY LCA GROUPS
140
120
100
80
60
Low Risk
Overweigh Moderate risk
Overweight High Risk
Obese High Risk
LCA

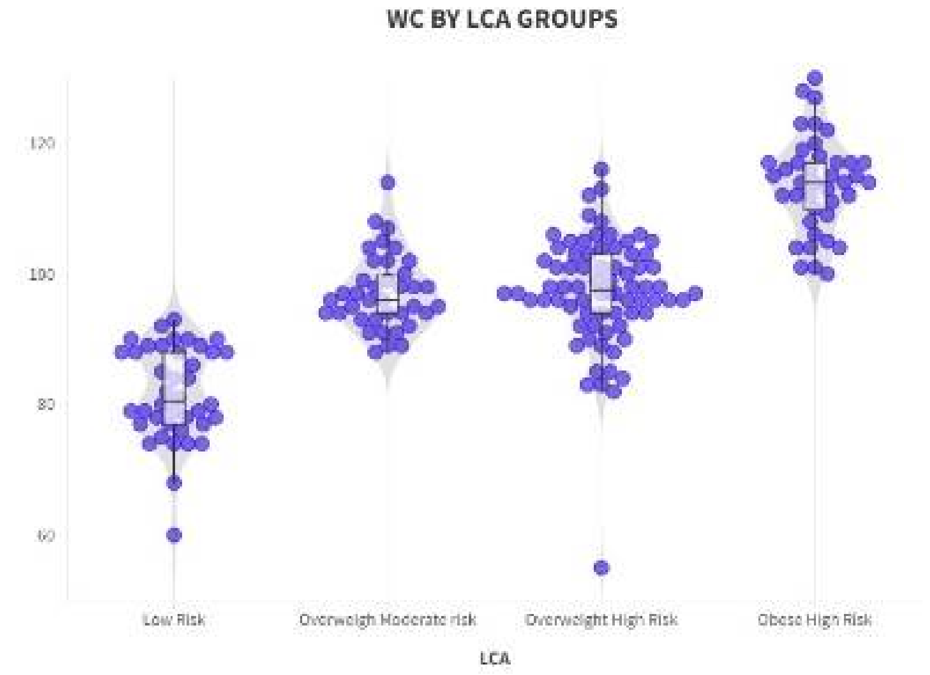
WC BY LCA GROUPS
120
100
80
60
Low Risk
Overweigh Moderate risk
Overweight High Risk
Obese High Risk
LCA

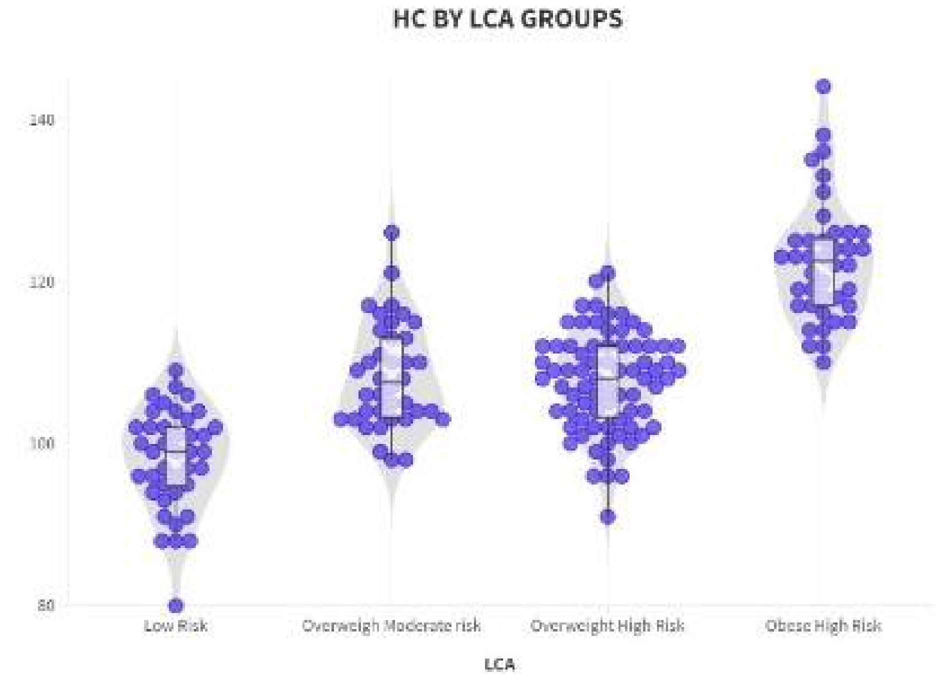
HC BY LCA GROUPS
140
120
100
80
Low Risk
Overweigh Moderate risk
Overweight High Risk
Obese High Risk
LCA

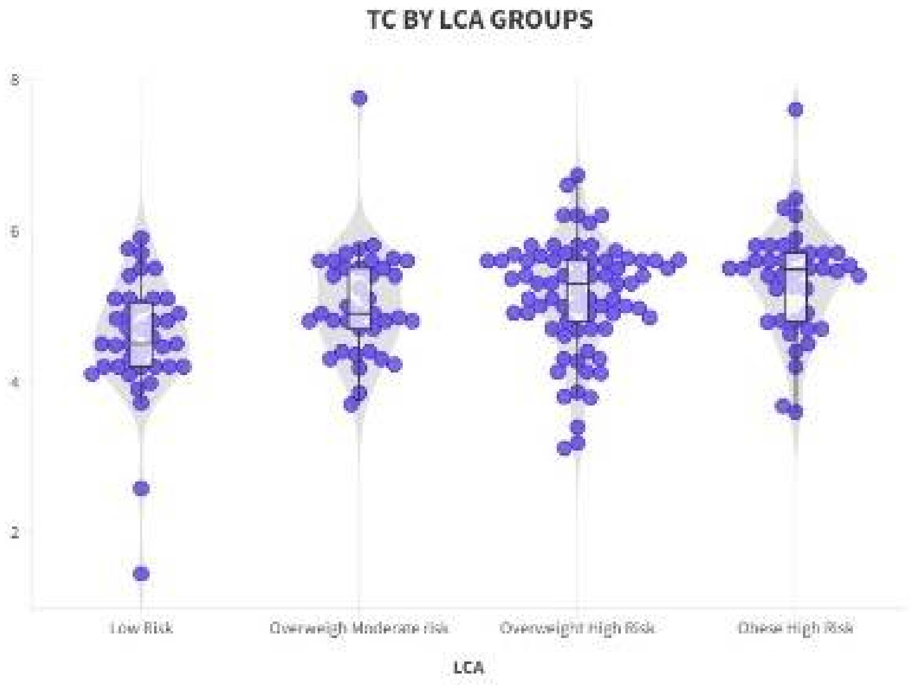
TC BY LCA GROUPS
8
6
4
2
Low Risk
Overweigh Moderate risk
Overweight High Risk
Obese High Risk
LCA

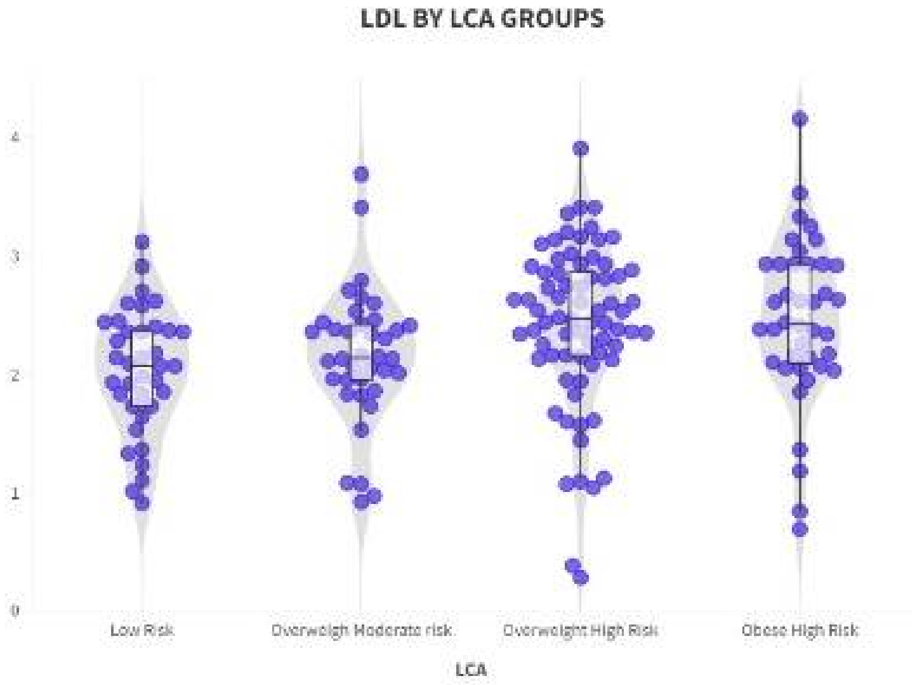
LDL BY LCA GROUPS
4
3
2
1
0
Low Risk
Overweigh Moderate risk
Overweight High Risk
Obese High Risk
LCA

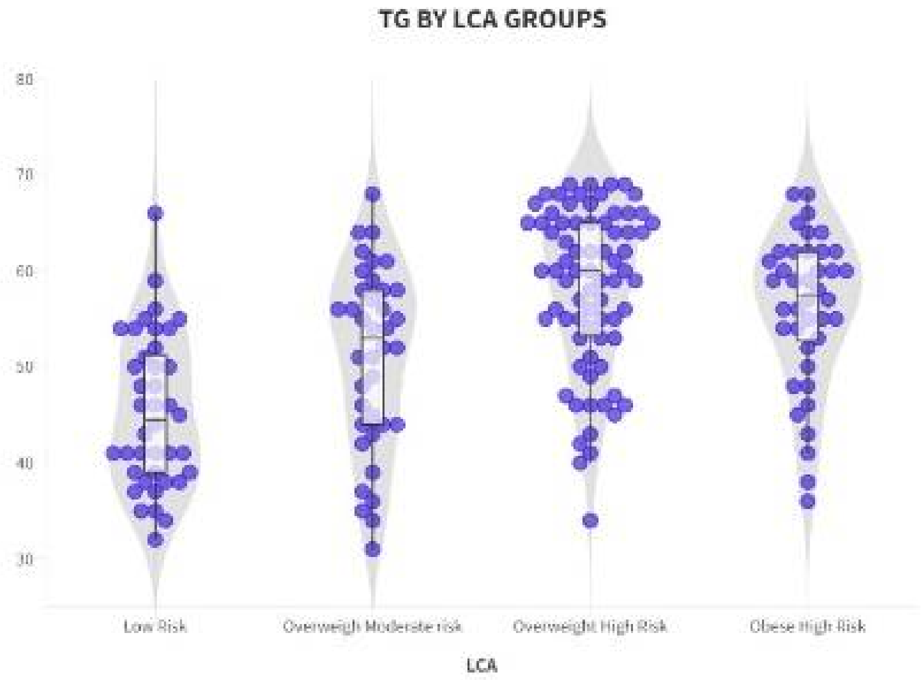

LCA: Latent Class Analysis; BMI: Body Mass Index; WC: Waist circumference; HC: Hip circumference; SBP: Systolic Blood Pressure; DBP: Diastolic Blood Pressure; TC: Total cholesterol; LDL: Low-density lipoprotein cholesterol; HDL: High-density lipoprotein cholesterol; TG: Triglycerides.
